# Carbapenem Resistance in Gram-Negative Bacteria: The Not-So-Little Problem in the Little Red Dot

**DOI:** 10.3390/microorganisms4010013

**Published:** 2016-02-16

**Authors:** Jocelyn Qi Min Teo, Yiying Cai, Tze-Peng Lim, Thuan Tong Tan, Andrea Lay-Hoon Kwa

**Affiliations:** 1 Department of Pharmacy, Singapore General Hospital, Outram Road, Singapore 169608, Singapore; jocelyn.teo.q.m@sgh.com.sg (J.Q.M.T.); cai.yiying@sgh.com.sg (Y.C.); lim.tze.peng@sgh.com.sg (T.-P.L.); 2 Department of Pharmacy, Faculty of Science, National University of Singapore, Block S4A, Level 3, 18 Science Drive 4, Singapore 117543, Singapore; 3 Office of Clinical Sciences, Duke-National University of Singapore Medical School, 8 College Road, Singapore 169857, Singapore; 4 Department of Infectious Diseases, Singapore General Hospital, Outram Road, Singapore 169608, Singapore; tan.thuan.tong@sgh.com.sg; 5 Emerging Infectious Diseases, Duke-National University of Singapore Medical School, 8 College Road, Singapore 169857, Singapore

**Keywords:** extensively-drug resistant, molecular epidemiology, carbapenemase, infection control, antibiotic combinations

## Abstract

Singapore is an international travel and medical hub and faces a genuine threat for import and dissemination of bacteria with broad-spectrum resistance. In this review, we described the current landscape and management of carbapenem resistance in Gram-negative bacteria (GNB) in Singapore. Notably, the number of carbapenem-resistant Enterobacteriaceae has exponentially increased in the past two years. Resistance is largely mediated by a variety of mechanisms. Polymyxin resistance has also emerged. Interestingly, two *Escherichia coli* isolates with plasmid-mediated *mcr*-1 genes have been detected. Evidently, surveillance and infection control becomes critical in the local setting where resistance is commonly related to plasmid-mediated mechanisms, such as carbapenemases. Combination antibiotic therapy has been proposed as a last-resort strategy in the treatment of extensively drug-resistant (XDR) GNB infections, and is widely adopted in Singapore. The diversity of carbapenemases encountered, however, presents complexities in both carbapenemase detection and the selection of optimal antibiotic combinations. One unique strategy introduced in Singapore is a prospective *in vitro* combination testing service, which aids physicians in the selection of individualized combinations. The outcome of this treatment strategy has been promising. Unlike countries with a predominant carbapenemase type, Singapore has to adopt management strategies which accounts for diversity in resistance mechanisms.

## 1. Introduction

Broad-spectrum antimicrobial resistance, where resistance to multiple, or even all available antibiotic classes, is a key global healthcare problem [1]. In Asia, the problem has gone beyond extended-spectrum β-lactamase (ESBL) production [2]. As a consequence of tremendous carbapenem usage in ESBL-endemic settings, emerging resistance trend to carbapenems is now a major concern [3]. In a recent global surveillance report by the World Health Organisation (WHO), carbapenem resistance rates in *Klebsiella* species, one of the nine bacteria of international concern, has now reached the excess of 50% in the South East Asian region [4]. 

Currently, long-standing collaborative regional surveillance of multi-drug resistant organisms (MDROs) is in place in Europe and the Americas. Unfortunately, such intensive surveillance efforts appear to be lacking in the Southeast Asian region. In the recent WHO surveillance report, less than half of the Southeast Asian member states returned datasets [4]. In Singapore, it is recognized that surveillance is integral to the control of MDROs. In response to the rising global MDRO trends, a national MDRO program with escalated surveillance activities has been established [5]. Furthermore, our status as an international travel and medical hub, together with the established healthcare infrastructure, positions Singapore as a suitable antimicrobial surveillance sentinel site for the region. In this paper, we aim to provide an update to the current landscape of broad-spectrum antibiotic resistance in key Gram-negative organisms in Singapore, focusing on the epidemiology and management of carbapenem-resistant *Acinetobacter* species, *Pseudomonas aeruginosa*, and Enterobacteriaceae species in the local acute care setting.

## 2. Epidemiology

The epidemiology of resistance in Gram-negative bacteria (GNB) can vary considerably by geographical locations [4]. Singapore has a racially- and culturally-diverse population, comprising a large migrant population. The country’s strategic geographical location at international crossroads also makes it an international hub for travel, trade, and medical tourism. The diversity in the epidemiology of broad-spectrum resistant GNB in our setting is likely the consequence of this exchange of highly-mobile populations.

### 2.1. Prevalence

In 2006, a laboratory-based surveillance program was organized by an informal network of infectious disease professionals in Singapore to gain an insight to the MDRO problem. This first comprehensive national survey, conducted in 2006–2008, detected imipenem resistance rates of 46.2% and 7.5% in *Acinetobacter baumannii* and *Pseudomonas aeruginosa*, respectively [6]. In this period, carbapenem resistance in Enterobacteriaceae species was rare or non-existent [7]. Owing to the endemicity of ESBLs in our setting and the corresponding increasing carbapenem usage, it is not surprising that we -begun to witness a gradual increase in the number of these carbapenem-resistant Enterobacteriaceae (CRE) isolates [8]. After this survey, there are no published data for resistance rates of these organisms. 

**Figure 1:**
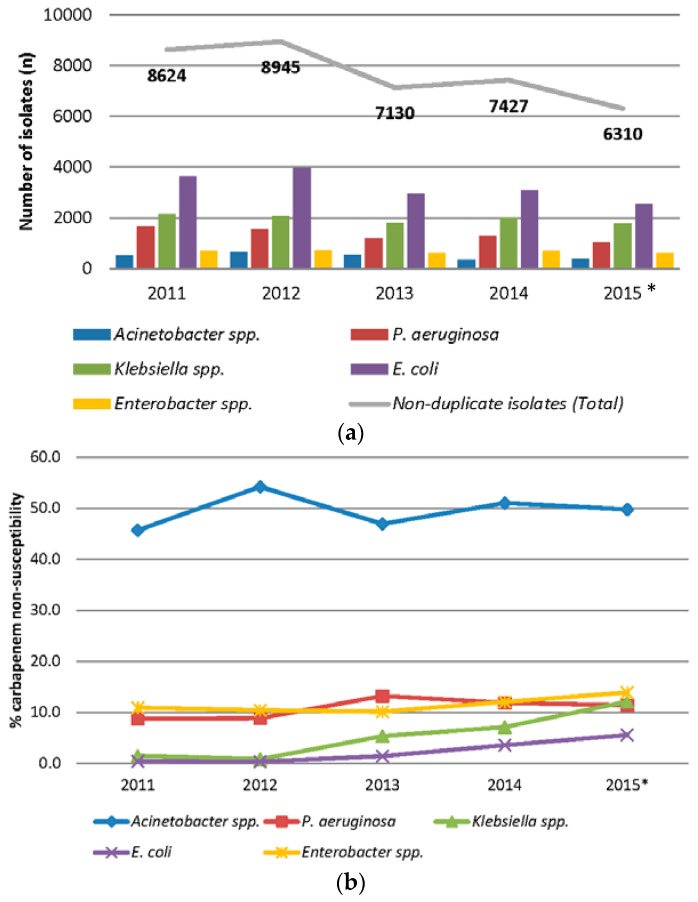
(**a**) Number of non-duplicate key Gram-negative pathogens in Singapore General Hospital from 2011 to 2015; * includes data from the first 10 months; and (**b**) Carbapenem non-susceptibility rates (% intermediate and resistant) of non-duplicate key Gram-negative pathogens in Singapore General Hospital from 2011 to 2015; * includes data from the first 10 months.

A review of in-house data from the Singapore General Hospital, the largest local tertiary acute care hospital with more than 1900 beds, indicated that imipenem resistance rates remained highest in *Acinetobacter* spp. among non-duplicate clinical isolates collected in 2011–2015 (Figure 1a,b) [9]. Screening cultures were not included in this survey, as active surveillance of CRE was only initiated in 2013. Imipenem resistance rates for *Acinetobacter* spp. and *Pseudomonas aeruginosa* remained relatively stable over the study period and have crept up slightly to an average of 50.5% and 10.2% compared to the 2008 survey [6]. More importantly, carbapenem resistance has emerged in Enterobacteriaceae, and the numbers are rapidly increasing in *Klebsiella* and *Enterobacter* species. 

A recent national survey of local carbapenemase-producing (CP) CRE isolates suggested that the magnitude of carbapenem resistance in Enterobacteriaceae is more marked than it appears [10]. Between 2010 and 2014, more than 1003 carbapenemase-producing Enterobacteriaceae (CPE) isolates were submitted to the National Public Health Laboratory by local hospitals. While the exponential increase in numbers in the last two years (>70% of surveyed isolates occurred in this period) might be related to increased routine surveillance screening, there certainly is a concern for this increasing reservoir of (CPE) colonizers. High counts of CP-CRE were also easily recovered from the sewage systems of four hospitals here, suggesting that screening is critical in our setting to limit any potential transmission [11]. 

Typically, carbapenem-resistant Gram-negative infections still remain highly susceptible to antibiotics such as tigecycline and polymyxins. Unfortunately, polymyxin and tigecycline resistance has been detected in local carbapenem-resistant Gram-negative clinical isolates. It is disconcerting that close to 20% of 100 carbapenem-resistant *Klebsiella* spp. clinical isolates were found to be polymyxin B- and/or tigecycline-resistant in a recent survey of carbapenem-resistant *Klebsiella spp*. (Table 1) [12]. Out of these polymyxin- or tigecycline-resistant isolates, 80% were carbapenemase producers.

**Table 1:** Antibiotic susceptibilities of 100 carbapenem-resistant *Klebsiella* spp. isolates.

Antibiotic	MIC_50_ (µg/mL)	MIC_90_ (µg /mL)	Range (µg/mL)	%S
Ertapenem	≥32	≥32	1 - ≥32	0
Imipenem	16	≥32	0.5 - ≥32	8.1
Meropenem	≥32	≥32	0.5 - ≥32	6.6
Doripenem	16	≥32	0.5 - ≥32	5.1
Amikacin	4	≥128	≤1 - ≥128	75.7
Aztreonam	≥128	≥128	≤0.5 - ≥128	2.9
Cefepime	≥64	≥64	≤1 - ≥64	2.2
Levofloxacin	16	≥64	≤ 0.25 - ≥64	34.6
Piperacillin/tazobactam	≥256	≥256	8 - ≥256	1.5
Polymyxin B	1	16	≤0.25 - ≥32	83.1
Tigecycline	1	4	≤0.25 - 16	82.4

### 2.2. Mechanisms of Resistance

Broad-spectrum antibacterial resistance is related to intrinsic and/or multiple acquired resistance mechanisms inclusive of penicillin-binding proteins (PBPs) production, enzymatic mechanisms of drug modification, mutated drug targets, enhanced efflux pump expression, and altered membrane permeability [13]. It is highly disconcerting that β-lactamases such as carbapenemases, once rare, are now extensively being reported across the globe, especially in Enterobacteriaceae [14]. The genes encoding these carbapenemases are often housed together with other genes encoding resistance to non-β-lactam antibiotics on highly-mobile plasmids, leading to rapid transmission of broad-spectrum resistance [15]. 

Major groups of CPE have been described in Singapore. It is reported that almost 40% of carbapenem-resistant clinical *Klebsiella* spp. isolates in Singapore tested positive for a carbapenemase [16]. A more recent survey revealed that 86% of carbapenem-resistant *Klebsiella* spp. isolates from Singapore General Hospital harbours at least one carbapenemase [12]. *bla*_IMP-1_ in *K. pneumoniae* was first identified in Singapore in 1996 [17]. Shortly after the first *bla*_NDM-1_ was reported in an Indian patient in Sweden, NDM-positive *Klebsiella pneumoniae* was isolated from two patients who returned from India and Bangladesh in 2010 [18]. Four *bla*_KPC-2_-positive *K. pneumoniae* isolates were identified in patients from two different hospitals in Singapore the following year [19]. More recently, *bla*_OXA-48-like_-producers have also been described [20]. Uncommon carbapenemases such as *bla*_IMI-1_ and *bla*_OXA-232_ have been detected in *Enterobacter cloacae* and *K. pneumonia*, respectively [21,22]. 

Koh *et al.* published one of the first systematic surveys of carbapenemase genes in Singapore. In this report, majority of the CP-CRE isolates from Singapore General hospital carried *bla*_NDM_ [8]. Likewise, a national survey of seven hospitals from 2010–2012 reported *bla*_NDM_-producers as the dominant carbapenemase-producer (64%), followed by *bla*_OXA-181_-producers (25%) in Enterobacteriaceae. *bla*_KPC_- and *bla*_IMP/VIM_-producers were less common [16]. However, there seems to be a changing trend in the types of carbapenemase producers in the past two years. In 2013, the number of *bla*_KPC_-producers exponentially increased, and have overtaken the *bla*_NDM_-producers as the dominant type in 2014 [10]. There was also an emergence of carbapenemase co-producers, primarily *bla*_OXA-181_ with b*la*_NDM_ [12,23]. Carbapenem resistance in *Acinetobacter* species in Singapore is largely associated with IS*Aba1*-*bla*_OXA-23-like_ [24]. *bla*_IMP-4_ and *bla*_OXA-58-like_ have also been described to a lesser extent in local *Acinetobacter* spp. isolates [24,25]. Carbapenemase production is less common in *P. aeruginosa*, but *bla*_IMP_ and *bla*_VIM_ have been described in isolates here [26,27,28]. It is likely that non-carbapenemase-mediated mechanisms accounted for carbapenem resistance in our local *P. aeruginosa* isolates.

Carbapenem-resistant *A. baumannii* isolates in Singapore primarily belonged to the international clones (IC) I and II [29]. Data obtained from a Singapore General Hospital’s archived repository identified three outbreak clones (IC I , IC II, and IC2 II, respectively) from the three outbreaks in 1996, 2001, and 2006. These clones harbored *bla*_OXA-64_, *bla*_OXA-66_, and *bla*_OXA-51-like_, respectively. The phenomenon is consistent with reports from China and Korea where these international clones have shown to have outbreak potential [30]. Local carbapenem-resistant *P. aeruginosa* isolates did not belong to any major international clonal complexes such as ST111 and ST235 [31]. Unlike in North America, Israel, and Europe, where *K. pneumoniae* ST258 is highly prevalent, the local *K. pneumoniae* appeared to be of highly varied STs (ST 11, 14, 15, 17, 29, 42, 48, 147, 163, 231, 237, 273, 437, 568, 841, and 885) [8,16,19,23,32,33]. 

Recently, a study reported the emergence of the *mcr*-1 gene in China, which was associated with transmissible, plasmid-mediated colistin resistance. It was suggested that *mcr*-1 might have spread to Southeastern Asia [34]. While the research in carbapenem resistance mechanisms has been actively ongoing, work on the molecular determinants of polymyxin resistance has been sparse. One study related chromosomal mutations in *lpx* and *pmr*B genes to polymyxin B resistance in local *A. baumannii* isolates [35]. There has yet to be any reports of *mcr*-1 in Singapore. Reviewing 17 whole genome datasets of polymyxin B-resistant carbapenem-resistant Enterobacteriaceae available in Singapore General Hospital, we detected the presence of *mcr-1* in two *E. coli* isolates (one from stool and one from wound tissue), both with polymyxin B MICs of 4 µg/mL [36]. Further research can shed light on the role of plasmid-mediated transmission of polymyxin resistance in the local context. 

## 3. Control and Prevention

The control of MDRO has been designated as a national priority in Singapore. The Ministry of Health (MOH), Singapore, provides leadership and coordinates MDRO control strategies among healthcare facilities. Key efforts include the establishment of a national MDRO program and publication of guidelines for the control and prevention of MDRO [37]. In these guidelines, the MOH has recommended a multi-pronged management approach through a MDRO bundle comprising active surveillance, antimicrobial management (antimicrobial stewardship programs), isolation precautions, and hand and environmental hygiene.

### 3.1. Infection Control and Active Surveillance

Active surveillance and vigilant infection control are critical in controlling the spread of these resistant GNBs which have a high propensity for transmission. Routine screening for CP-CRE is recommended in all acute care facilities in Singapore. Screening criteria differs between hospitals, but in general includes all patients with either the presence of risk factors (institution-specific) or history of hospitalization overseas or locally in a private hospital within the past year. 

Currently, there is no standardized carbapenemase detection method and much research is ongoing to identify more accurate, timely, and cost-effective methods. In general, carbapenemases are detected via: (1) phenotypic methods (modified Hodge test, inhibitor-based tests, or selective chromogenic screening media; (2) analytical and biochemical detection methods (spectrophotometry, MALDI-TOF, Carba NP test, *etc.*); and (3) molecular methods (PCR and sequencing) [38,39]. The accuracy (sensitivity/specificity), costs, and ease of use vary among the different methods. Molecular methods remain the gold standard in carbapenemase detection, as it is relatively fast, especially if real-time PCR technology is employed, and offer excellent sensitivities and specificities. It is also the method used to identify the specific carbapenemase involved [40]. The cost and need for trained experts, however, prohibit its use in many routine laboratories. In Singapore, carbapenemase detection via PCR is mostly limited to epidemiological surveillance and research purposes, and primarily performed at the National Public Health Laboratory. 

In the routine laboratories in Singapore, surveillance of rectal/stool cultures are screened for carbapenemase production using selective media, e.g., ChromID^®^ CARBA SMART plates (bioMérieux SA, Marcy-l'Etoile, France). Different selective media have different performance in the identification of different carbapenemases or different species [41]. In general, these media are better in the detection of CPEs with high-level resistance (KPC and metallo-β-lactamases) [15]. In addition, results may be affected by non-carbapenemase-producing isolates or the presence of other mechanisms, like AmpC/ESBL production. In view of the diversity of CPEs in the local context, the use of ChromID^®^ CARBA SMART plates, which combines two selective media, was selected to aid the identification of OXA-48-producers. 

CPE-positive screening isolates and clinical CRE isolates are confirmed with the Carba NP test and/or modified Hodge test. The rapid, user-friendly Carba NP test offers high sensitivity and specificity [42]. However, false negative results with OXA-48-producers have been reported [43]. The modified Hodge test is the CLSI-proposed test for the confirmation of putative carbapenemase producers [44], although it has a lower sensitivity for metallo-β-lactamases detection [45] and is impacted by the type of test carbapenem used [46], species involved [38], and presence of AmpC and ESBL [47]. Therefore, this test is mostly used as a supplementary test. Some local laboratories utilize inhibition-based carbapenamses detection test for further confirmation in cases of doubtful results. In the context of Singapore, the endemicity of ESBLs/AmpCs, relatively high rate of OXA-48-producers, and lack of resources for molecular techniques pose a challenge in carbapenemase detection. Unfortunately, until a low-cost PCR assay, which can be easily integrated into the local routine laboratories’ infrastructure, is readily available, we have to rely on a combination of phenotypic and biochemical methodsfor detection.

Patients with positive MDROs (inclusive of MDR *A. baumannii*, MDR *P. aeruginosa*, and CP-CRE) are isolated in single rooms, if available, or cohorted in designated wards. In addition, all CP-CRE cases are notified to the MOH and cultures are sent to the National Public Health Laboratory, which is tasked to monitor the molecular epidemiology of MDROs. Hospitals also periodically report MDRO rates to the MOH.

### 3.2. Antimicrobial Stewardship Programs (ASP) in Singapore

Since 2008, multi-disciplinary ASPs have been implemented to promote judicious and appropriate antimicrobial prescribing in Singapore. In this respect, we can be considered a forerunner in ASP among Southeast Asian countries. In 2011, the government-funded National ASP was implemented in all public hospitals in Singapore [48,49,50,51]. The hospitals employ a multi-pronged strategy including a prospective audit and feedback approach, formulary restrictions, development of antimicrobial use guidelines, and use of computerized clinical decision support systems [48,52,53]. While antibiotics reviewed by the ASP in each hospital differ, depending on the choice of work-horse antibiotics and the consumption trend in the hospital, antibiotics with broad-spectrum activity against GNB (e.g., piperacillin/tazobactam, carbapenems) were included for review in all hospitals. On a periodic basis, key performance indicators of the program, such as acceptance rates of ASP interventions and patient safety outcomes, are submitted to the MOH as a mandatory requirement. 

Several local institutions have published one or more studies describing their ASP experience [48,49,51,53,54,55,56]. In these published studies, antibiotic appropriateness post-ASP implementation ranged from 66% to 78%, and acceptance of ASP interventions ranged from 61% to 87%. Implementation of ASP resulted in a significant reduction of consumption of one or more antibiotics, without compromising patient safety [48,53,54]. In fact, one study demonstrated that acceptance of ASP interventions was associated with a reduction in the length of stay and lower 30-day re-admission and re-infection rates [54]. The same group of authors also demonstrated that early interventions within 48 h of antibiotic initiation, performed even before culture results are available, could reduce duration of antibiotic therapy without compromising patients’ safety [55]. 

To date, none of the studies demonstrated reversal of antimicrobial resistance. However, in a recent study where the authors explored the outcomes of carbapenem de-escalation by ASP, a significantly lower incidence of carbapenem-resistant *A. baumannii* was observed in patients where carbapenem de-escalation occurred [49]. Overall, ASP in Singapore has shown promise with respect to reducing consumption of broad-spectrum antimicrobials, in particular the carbapenems. We are hopeful that with continued efforts, a reduction in antimicrobial resistance amongst GNB will be achieved.

## 4. Treatment

### Use of Antibiotic Combination Therapy against XDR-GNB

Given the current resistance landscape, physicians in Singapore face a severe lack of effective antimicrobial therapies to treat infections caused by resistant GNB organisms. While polymyxin B, an old antibiotic once forsaken due to an allegedly high rate of toxicities, has been resurrected for clinical use, the presence of polymyxin B heteroresistance has limited its utility as a monotherapy in severe or deep-seated infections [57,58]. In recent years, the use of antibiotic combination therapy has been proposed by experts as the best practice in the management of infections by these organisms, and this strategy has been increasingly employed by physicians in Singapore [59]. Unfortunately, to date there is no definitive scheme to guide selection of antibiotic combinations against drug-resistant GNB. Morrill and colleagues has proposed a potential algorithm for the treatment of CRE infections [60]. The algorithm allowed dosing recommendations to be made by taking into consideration the site of infection and the corresponding empiric and antimicrobial susceptibility-directed treatment options. They concluded that optimization of dosing regimens of existing agents and combination therapy may be the most appropriate strategy. Zavascki *et al.* proposed a treatment flowchart for choosing antimicrobial agents for combination therapy against Gram-negative bacterial infections [59]. The strategy is based on the selection of a cornerstone antibiotic in combination with an adjuvant antibiotic to maximize bacterial killing and minimize emergence of resistance. There is growing evidence, although mostly were almost exclusively derived from non-randomized studies, suggesting that antibiotic combination therapy may offer a relative advantage over monotherapy with respect to reducing mortality in the critically ill population infection with CRE [61]. In a multi-center, randomized, controlled trial comparing colistin *versus* colistin with rifampicin for the treatment of XDR carbapenem-resistant *A. baumannii* infections in critically-ill patients, the addition of rifampicin to colistin did not improve the primary outcome of 30-day mortality, although there is a significant increase in microbiological eradication in the combination arm, suggesting a potential benefit of colistin in combination with rifampicin for the treatment of XDR *A. baumannii* [62]. 

Currently, there have been a number of published studies describing the activity of multiple antibiotic combinations *in vitro* against drug-resistant GNB strains isolated in Singapore [63,64,65,66]. Against *A. baumannii*, polymyxin B plus rifampicin or tigecycline appeared to be most promising in *in vitro* time-kill studies, while against *P. aeruginosa*, triple-antibiotic combinations (amikacin plus meropenem/rifampicin plus polymyxin) were most promising [63,64,65]. *In vitro* combinations which were bactericidal against *K. pneumoniae* were highly varied [66]. The variety in effective combinations could potentially be related to the diverse clonal types and resistance mechanisms in local Enterobacteriaceae isolates. Overall, the results of these published studies somewhat acted as a guide for physicians to select empiric antibiotic combinations against local drug-resistant GNB strains. However, it should be noted that these combinations are not ubiquitously bactericidal against all local strains, and may result in treatment failure and selective amplification of further resistance [66]. 

In view of physicians’ increasing demand for guidance in the selection of antibiotic combinations, an *in vitro* antibiotic combination testing service was implemented in Singapore. Briefly, *in vitro* testing for up to 120 different antibiotic combinationsis performed prospectively using a modified time-kill method, with a turn-around time of 48 h [67]. The antibiotic combination panel was chosen based on the availability in our hospitals’ pharmacies. Each antibiotic was combined with one or more partner antibiotics from various antibiotic classes with different mechanism of actions, resulting in up to 120 different permutations [68]. Based on the *in vitro* results, a trained infectious disease pharmacist would then recommend the most optimal combination, taking into account the patient’s clinical characteristics, site of infection, and the PK/PD parameter for efficacy of each antibiotic. Combination testing is performed only on XDR-GNB, on the premise that conventional susceptibility testing provided little or no information to guide antibiotic selection, and that selection of antibiotic combination based on results from *in vitro* testing is preferable to that of the individual physician’s preference. 

We have previously described *in vitro* results of the prospective combination testing service, as well as clinical outcomes of patients who received *in vitro* combination testing guided therapy [69]. From 2009 to 2015, a total of 50 patients received *in vitro* combination testing guided therapy. The most common GNB sent for combination testing was *P. aeruginosa*, followed by *A. baumannii* and *K. pneumoniae*. No combination was universally effective against each GNB species *in vitro*, emphasizing the strain-specific nature of the effective combinations. Interestingly, combinations that were bactericidal *in vitro* appeared to be related to the underlying mechanisms of resistance. High rates of clinical resolution (83%) and relatively low 30-day in-hospital mortality rates (14%) were observed. Based on the results of this preliminary study, it appears that until alternative strategies are to effectively treat drug-resistant GNB infections, the use of antibiotic combinations, guided by *in vitro* combination testing, is a feasible option in our local setting. However, further clinical studies will be required to fully evaluate the utility of this service. 

## 5. Conclusions

Antimicrobial resistance is a global public threat that requires a concerted effort from all stakeholders. Owing to our geographical location, the local molecular epidemiology is highly diverse. Unlike countries in the Americas and Europe, where a predominant clonal or resistance type is observed, this diversity presents complexities in infection control and management strategies. The high carbapenemase occurrence rates underscore the critical need for an effective, timely surveillance program to limit the spread. Surveillance, together with optimizing the use of currently available antibiotics through antimicrobial stewardship must be continued as part of the containment efforts. However, we need to identify more rapid, low-cost, and accurate methods suitable for carbapenemase detection in our ESBL-endemic setting. Unique strategies, which take into consideration the diversity in molecular epidemiology, such as strain-specific *in vitro* combination testin, as a means to devise appropriate therapeutic options in a clinically-relevant manner for XDR-GNB infections, should be explored further. 

## References

[1] Paterson D.L., Doi Y. (2007). A step closer to extreme drug resistance (XDR) in Gram-negative bacilli. Clin. Infect. Dis. Off. Pub. Infect. Dis. Soc. Am..

[2] Jean S.S., Hsueh P.R. (2011). High burden of antimicrobial resistance in Asia. Int. J. Antimicrob. Agents.

[3] Hawkey P.M. (2015). Multidrug-resistant Gram-negative bacteria: A product of globalization. J. Hosp. Infect..

[4] World Health Organization Antimicrobial Resistance: Global Report on Surveillance. http://apps.who.int/iris/bitstream/10665/112642/1/9789241564748_eng.pdf.

[5] Molton J.S., Tambyah P.A., Ang B.S., Ling M.L., Fisher D.A. (2013). The global spread of healthcare-associated multidrug-resistant bacteria: A perspective from Asia. Clin. Infect. Dis. Off. Publ. Infect. Dis. Soc. Am..

[6] Hsu L.Y., Tan T.Y., Tam V.H., Kwa A., Fisher D.A., Koh T.H. (2010). Network for Antimicrobial Resistance Surveillance (Singapore). Surveillance and correlation of antibiotic prescription and resistance of Gram-negative bacteria in Singaporean hospitals. Antimicrob. Agents Chemother..

[7] Tan T.Y., Hsu L.Y., Koh T.H., Ng L.S., Tee N.W., Krishnan P., Lin R.T., Jureen R. (2008). Antibiotic resistance in Gram-negative bacilli: A Singapore perspective. Ann. Acad Med. Singap..

[8] Koh T.H., Cao D., Shan Q.Y., Bacon A., Hsu L.Y., Ooi E.E. (2013). Acquired carbapenemases in enterobactericeae in Singapore, 1996–2012. Pathology.

[9] Teo J., Lim T.P., Cai Y., Koh T.H., Tan T.T., Koh T.H., Kwa A.L. (2016). Molecular epidemiology of carbapenem-resistant Gram negative bacteria in Singapore. Antimicrob. Agents Chemother..

[10] Krishnan P.U., La M.V., Jureen R., Koh M., Teo J., Lin R. Changing trends in carbapenemase-producing Enterobacteriaceae in Singapore from 2010 to 2014 (August). Proceedings of the 25th European Congress of Clinical Microbiology and Infectious Diseases.

[11] Koh T.H., Ko K., Jureen R., Deepak R.N., Tee N.W., Tan T.Y., Tay M.R., Lee V.J., Barkham T.M. (2015). High counts of carbapenemase-producing Enterobacteriaceae in hospital sewage. Infect. Control. Hosp. Epidemiol..

[12] Teo J., Lee S., Leck H., Chia S., Wang R., Cai Y., Lim T.P., Koh T.H., Tan T.T., Kwa A.L. Molecular characterization of carbapenem-resistant *Klebsiella* species in Singapore. Proceedings of the 25th European Congress of Clinical Microbiology and Infectious Diseases.

[13] Alekshun M.N., Levy S.B. (2007). Molecular mechanisms of antibacterial multidrug resistance. Cell.

[14] Nordmann P., Poirel L. (2014). The difficult-to-control spread of carbapenemase producers among Enterobacteriaceae worldwide. Clin. Microbiol. Infect. Off. Publ. Eur. Soc. Clin. Microbiol. Infect. Dis..

[15] Nordmann P., Naas T., Poirel L. (2011). Global spread of carbapenemase-producing Enterobacteriaceae. Emerg. Infect. Dis..

[16] Balm M.N., Ngan G., Jureen R., Lin R.T., Teo J.W. (2013). Oxa-181-producing *Klebsiella pneumoniae* establishing in Singapore. BMC Infect. Dis..

[17] Koh T.H., Babini G.S., Woodford N., Sng L.H., Hall L.M., Livermore D.M. (1999). Carbapenem-hydrolysing IMP-1 beta-lactamase in *Klebsiella pneumoniae* from Singapore. Lancet.

[18] Koh T.H., Khoo C.T., Wijaya L., Leong H.N., Lo Y.L., Lim L.C., Koh T.Y. (2010). Global spread of New Delhi metallo-beta-lactamase 1. Lancet Infect. Dis..

[19] Balm M.N., Ngan G., Jureen R., Lin R.T., Teo J. (2012). Molecular characterization of newly emerged *bla*_KPC_-2-producing *Klebsiella pneumoniae* in Singapore. J. Clin. Microbiol..

[20] Marimuthu K., Teo J.W., Fong P.B., Chin J.O., Qi K.J., Boon D.L., Ping A.C., Krishnan P., Peng B.A. (2014). First report of emergence of OXA-48 carbapenemase-producing Enterobacteriaceae in Singapore: Proactive or reactive infection control strategy?. Am. J. Infect. Control..

[21] Teo J.W., La M.V., Krishnan P., Ang B., Jureen R., Lin R.T. (2013). Enterobacter cloacae producing an uncommon class a carbapenemase, IMI-1, from Singapore. J. Med. Microbiol..

[22] Teo J.W., Kurup A., Lin R.T., Hsien K.T. (2013). Emergence of clinical *Klebsiella pneumoniae* producing OXA-232 carbapenemase in Singapore. New Microbes New Infect..

[23] Balm M.N., La M.V., Krishnan P., Jureen R., Lin R.T., Teo J.W. (2013). Emergence of *Klebsiella pneumoniae* co-producing NDM-type and OXA-181 carbapenemases. Clin. Microbiol. Infect. Off. Publ. Eur. Soc. Clin. Microbiol. Infect. Dis..

[24] Koh T.H., Sng L.H., Wang G.C., Hsu L.Y., Zhao Y. (2007). IMP-4 and oxa beta-lactamases in *Acinetobacter baumannii* from Singapore. J. Antimicrob. Chemother..

[25] Koh T.H., Sng L.H., Wang G.C., Hsu L.Y., Zhao Y. (2007). Carbapenemase and efflux pump genes in acinetobacter calcoaceticus *Acinetobacter baumannii* complex strains from Singapore. J. Antimicrob. Chemother..

[26] Koh T.H., Wang G.C., Sng L.H. (2004). Clonal spread of IMP-1-producing *Pseudomonas aeruginosa* in two hospitals in Singapore. J. Clin. Microbiol..

[27] Koh T.H., Khoo C.T., Tan T.T., Arshad M.A., Ang L.P., Lau L.J., Hsu L.Y., Ooi E.E. (2010). Multilocus sequence types of carbapenem-resistant *Pseudomonas aeruginosa* in Singapore carrying metallo-beta-lactamase genes, including the novel *bla*_IMP-26_ gene. J. Clin. Microbiol..

[28] Koh T.H., Wang G.C., Sng L.H. (2004). IMP-1 and a novel metallo-beta-lactamase, VIM-6, in fluorescent pseudomonads isolated in Singapore. Antimicrob. Agents Chemother..

[29] Koh T.H. (2013). Acquired Carbapenemases in Gram-Negative Bacilli in Singapore. Ph.D. Thesis.

[30] Park Y.K., Lee G.H., Baek J.Y., Chung D.R., Peck K.R., Song J.H., Ko K.S. (2010). A single clone of *Acinetobacter baumannii*, ST22, is responsible for high antimicrobial resistance rates of *Acinetobacter* spp. Isolates that cause bacteremia and urinary tract infections in Korea. Microb. Drug Resist..

[31] Samuelsen O., Toleman M.A., Sundsfjord A., Rydberg J., Leegaard T.M., Walder M., Lia A., Ranheim T.E., Rajendra Y., Hermansen N.O. (2010). Molecular epidemiology of metallo-beta-lactamase-producing *Pseudomonas aeruginosa* isolates from norway and Sweden shows import of international clones and local clonal expansion. Antimicrob. Agents Chemother..

[32] Teo J., Ngan G., Balm M., Jureen R., Krishnan P., Lin R. (2012). Molecular characterization of NDM-1 producing Enterobacteriaceae isolates in Singapore hospitals. West. Pac. Surveill. Response J..

[33] Abate G., Koh T.H., Gardner M., Siu L.K. (2012). Clinical and bacteriological characteristics of *Klebsiella pneumoniae* causing liver abscess with less frequently observed multi-locus sequences type, ST163, from Singapore and Missouri, US. J. Microbiol. Immunol. Infect..

[34] Liu Y.Y., Wang Y., Walsh T.R., Yi L.X., Zhang R., Spencer J., Doi Y., Tian G., Dong B., Huang X. (2015). Emergence of plasmid-mediated colistin resistance mechanism MCR-1 in animals and human beings in China: A microbiological and molecular biological study. Lancet Infect. Dis..

[35] Lim T.P., Ong R.T., Hon P.Y., Hawkey J., Holt K.E., Koh T.H., Leong M.L., Teo J.Q., Tan T.Y., Ng M.M. (2015). Multiple genetic mutations associated with polymyxin resistance in *Acinetobacter baumannii*. Antimicrob. Agents Chemother..

[36] Teo J., Ong Rick T., Koh T.H., Lim T.P., Kwa A.L. (2016). Multidrug-resistant *Escherichia coli* co-harbouring *bla*_KPC_-2 and MCR-1 in Singapore. Lancet.

[37] Ministry of Health Guidelines for Control and Prevention of Multi-Drug Resistant Organisms (MDROs) in Healthcare Facilities. https://www.moh.gov.sg/content/dam/moh_web/Publications/Guidelines/Infection%20Control%20guidelines/GUIDELINES%20FOR%20CONTROL%20AND%20PREVENTION%20OF%20MULTI-DRUG%20RESISTANT%20ORGANISMS%20(MDROS)%20IN%20HEALTHCARE%20FACILITIES%20-%20Nov%202013.pdf.

[38] Nordmann P., Poirel L. (2013). Strategies for identification of carbapenemase-producing Enterobacteriaceae. J. Antimicrob. Chemother..

[39] Hammoudi D., Moubareck C.A., Sarkis D.K. (2014). How to detect carbapenemase producers? A literature review of phenotypic and molecular methods. J. Microbiol. Methods.

[40] Nordmann P., Gniadkowski M., Giske C.G., Poirel L., Woodford N., Miriagou V., European Network on Carbapenemases (2012). Identification and screening of carbapenemase-producing Enterobacteriaceae. Clin. Microbiol. Infect. Off. Publ. Eur. Soc. Clin. Microbiol. Infect. Dis..

[41] Hrabak J., Chudackova E., Papagiannitsis C.C. (2014). Detection of carbapenemases in Enterobacteriaceae: A challenge for diagnostic microbiological laboratories. Clin. Microbiol. Infect. Off. Publ. Eur. Soc. Clin. Microbiol. Infect. Dis..

[42] Nordmann P., Poirel L., Dortet L. (2012). Rapid detection of carbapenemase-producing Enterobacteriaceae. Emerg. Infect. Dis..

[43] Tijet N., Boyd D., Patel S.N., Mulvey M.R., Melano R.G. (2013). Evaluation of the carba np test for rapid detection of carbapenemase-producing Enterobacteriaceae and *Pseudomonas aeruginosa*. Antimicrob. Agents Chemother..

[44] Clinical and Laboratory Standards Institute (2010). Performance Standards for Antimicrobial Testing: Seventeenth Informational Supplement M100-S20.

[45] Girlich D., Poirel L., Nordmann P. (2012). Value of the modified hodge test for detection of emerging carbapenemases in Enterobacteriaceae. J. Clin. Microbiol..

[46] Miriagou V., Cornaglia G., Edelstein M., Galani I., Giske C.G., Gniadkowski M., Malamou-Lada E., Martinez-Martinez L., Navarro F., Nordmann P. (2010). Acquired carbapenemases in gram-negative bacterial pathogens: Detection and surveillance issues. Clin. Microbiol. Infect. Off. Publ. Eur. Soc. Clin. Microbiol. Infect. Dis..

[47] Pasteran F., Mendez T., Guerriero L., Rapoport M., Corso A. (2009). Sensitive screening tests for suspected class a carbapenemase production in species of Enterobacteriaceae. J. Clin. Microbiol..

[48] Teo J., Kwa A.L., Loh J., Chlebicki M.P., Lee W. (2012). The effect of a whole-system approach in an antimicrobial stewardship programme at the Singapore general hospital. Eur. J. Clin. Microbiol. Infect. Dis..

[49] Lew K.Y., Ng T.M., Tan M., Tan S.H., Lew E.L., Ling L.M., Ang B., Lye D., Teng C.B. (2015). Safety and clinical outcomes of carbapenem de-escalation as part of an antimicrobial stewardship programme in an esbl-endemic setting. J. Antimicrob. Chemother..

[50] Yeo C.L., Wu J.E., Chung G.W., Chan D.S., Chen H.H., Hsu L.Y. (2013). Antimicrobial stewardship auditing of patients reviewed by infectious diseases physicians in a tertiary university hospital. Antimicrob. Resist. Infect. Control..

[51] Seah X.F., Ong Y.L., Tan S.W., Krishnaswamy G., Chong C.Y., Tan N.W., Thoon K.C. (2014). Impact of an antimicrobial stewardship program on the use of carbapenems in a tertiary women's and children's hospital, Singapore. Pharmacotherapy.

[52] Chow A.L., Lye D.C., Arah O.A. (2015). Patient and physician predictors of patient receipt of therapies recommended by a computerized decision support system when initially prescribed broad-spectrum antibiotics: A cohort study. J. Am. Med. Inform. Assoc..

[53] Teng C.B., Ng T.M., Tan M.W., Tan S.H., Tay M., Lim S.F., Ling L.M., Ang B.S., Lye D.C. (2015). Safety and effectiveness of improving carbapenem use via prospective review and feedback in a multidisciplinary antimicrobial stewardship programme. Ann. Acad. Med. Singap..

[54] Liew Y.X., Lee W., Loh J.C., Cai Y., Tang S.S., Lim C.L., Teo J., Ong R.W., Kwa A.L., Chlebicki M.P. (2012). Impact of an antimicrobial stewardship programme on patient safety in Singapore general hospital. Int. J. Antimicrob. Agents.

[55] Liew Y.X., Lee W., Tay D., Tang S.S., Chua N.G., Zhou Y., Kwa A.L., Chlebicki M.P. (2015). Prospective audit and feedback in antimicrobial stewardship: Is there value in early reviewing within 48 h of antibiotic prescription?. Int. J. Antimicrob. Agents.

[56] Yeo C.L., Chan D.S., Earnest A., Wu T.S., Yeoh S.F., Lim R., Jureen R., Fisher D., Hsu L.Y. (2012). Prospective audit and feedback on antibiotic prescription in an adult hematology-oncology unit in Singapore. Eur. J. Clin. Microbiol. Infect. Dis..

[57] Cai Y., Lee W., Kwa A.L. (2015). Polymyxin b versus colistin: An update. Expert Rev. Anti Infect. Ther..

[58] Li J., Rayner C.R., Nation R.L., Owen R.J., Spelman D., Tan K.E., Liolios L. (2006). Heteroresistance to colistin in multidrug-resistant *Acinetobacter baumannii*. Antimicrob. Agents Chemother..

[59] Zavascki A.P., Bulitta J.B., Landersdorfer C.B. (2013). Combination therapy for carbapenem-resistant gram-negative bacteria. Expert Rev. Anti Infect. Ther..

[60] Morrill H.J., Pogue J.M., Kaye K.S., LaPlante K.L. (2015). Treatment options for carbapenem-resistant Enterobacteriaceae infections. Open Forum Infect. Dis..

[61] Falagas M.E., Lourida P., Poulikakos P., Rafailidis P.I., Tansarli G.S. (2014). Antibiotic treatment of infections due to carbapenem-resistant Enterobacteriaceae: Systematic evaluation of the available evidence. Antimicrob. Agents Chemother..

[62] Durante-Mangoni E., Signoriello G., Andini R., Mattei A., de Cristoforo M., Murino P., Bassetti M., Malacarne P., Petrosillo N., Galdieri N. (2013). Colistin and rifampicin compared with colistin alone for the treatment of serious infections due to extensively drug-resistant *Acinetobacter baumannii*: A multicenter, randomized clinical trial. Clin. Infect. Dis. Off. Publ. Infect. Dis. Soc. Am..

[63] Lim T.P., Tan T.Y., Lee W., Sasikala S., Tan T.T., Hsu L.Y., Kwa A.L. (2009). *In vitro* activity of various combinations of antimicrobials against carbapenem-resistant *Acinetobacter* species in Singapore. J. Antibiot. (Tokyo).

[64] Lim T.P., Lee W., Tan T.Y., Sasikala S., Teo J., Hsu L.Y., Tan T.T., Syahidah N., Kwa A.L. (2011). Effective antibiotics in combination against extreme drug-resistant *Pseudomonas aeruginosa* with decreased susceptibility to polymyxin b. PLoS ONE.

[65] Lim T.P., Tan T.Y., Lee W., Sasikala S., Tan T.T., Hsu L.Y., Kwa A.L. (2011). *In-vitro* activity of polymyxin B, rifampicin, tigecycline alone and in combination against carbapenem-resistant *Acinetobacter baumannii* in Singapore. PLoS ONE.

[66] Teo J., Lim T.P., Hsu L.Y., Tan T.Y., Sasikala S., Hon P.Y., Kwa A.L., Apisarnthanarak A. (2015). Extensively drug-resistant *Acinetobacter baumannii* in a thai hospital: A molecular epidemiologic analysis and identification of bactericidal polymyxin B-based combinations. Antimicrob. Resist. Infect. Control..

[67] Chua N.G., Lim T.P., Lee W., Liew Y., Teo J., Cai Y., Tan T.T., Kurup A., Kwa A.L. Combination bactericidal testing-guided therapy (MCBT-GT) is effective in the treatment of extensively drug-resistant (XDR) Gram-negative bacteria (GNB) infections. Proceedings of the Interscience Conference of Antimicrobial Agents and Chemotherapy.

[68] Lim T.P., Cai Y., Hong Y., Chan E.C., Suranthran S., Teo J.Q., Lee W.H., Tan T.Y., Hsu L.Y., Koh T.H. (2015). *In vitro* pharmacodynamics of various antibiotics in combination against extensively drug-resistant *Klebsiella pneumoniae*. Antimicrob. Agents Chemother..

[69] Cai Y., Teo J., Lim T.P., Leck H., Lee S., Chia S., Lee W., Tan T.T., Koh T.H., Kwa A.L. Correlating molecular mechanisms of resistance of extensively-drug resistant gram negative bacteria with *in vitro* activity of combination antibiotics & patient outcomes. Proceedings of the Interscience Conference of Antimicrobial Agents and Chemotherapy/International Congress of Chemotherapy and Infection.

